# The Low-Angle Boundaries Misorientation and Lattice Parameter Changes in the Root of Single-Crystalline CMSX-4 Superalloy Blades

**DOI:** 10.3390/ma14185194

**Published:** 2021-09-10

**Authors:** Robert Paszkowski, Włodzimierz Bogdanowicz, Dariusz Szeliga

**Affiliations:** 1 Institute of Materials Engineering, University of Silesia in Katowice, 1A 75 Pulku Piechoty St., 41-500 Chorzów, Poland; wlodzimierz.bogdanowicz@us.edu.pl; 2 Department of Materials Science, Rzeszów University of Technology, 2 Wincentego Pola St., 35-959 Rzeszów, Poland; dszeliga@prz.edu.pl

**Keywords:** CMSX-4 superalloys, low-angle boundaries, lattice parameter of γ′-phase

## Abstract

The relationship between the angles of misorientation of macroscopic low-angle boundaries (LABs) and changes in the lattice parameter of the γ′-phase around the LABs in the root of single-crystalline (SX) turbine blades made of CMSX-4 superalloy were studied. The blades with an axial orientation of the [001] type were solidified using an industrial Bridgman furnace with a 3 mm/min withdrawal rate. X-ray diffraction topography, the EFG Ω-scan X-ray diffraction method, scanning electron microscopy, and Laue diffraction were used to study the thin lamellar samples with a thickness of 0.5 mm and orientation of the surface perpendicular to the [001] direction. It is found that in the areas with a width of a few millimetres around LABs, decreases in the lattice parameter of the γ′-phase occur. These lattice parameter changes are related to the internal stresses of the γ′-phase caused by local changes in the concentration of alloying elements and/or to the dendrite bending near the LABs. X-ray topography used on two surfaces of thin lamellar samples coupled with the lattice parameter measurements of the γ′-phase near the LAB allows separating the misorientation component of LAB diffraction contrast from the component and visualising the internal stresses of the γ′-phase.

## 1. Introduction

Single-crystalline (SX) CMSX-4 turbine blades are used in the hot part of aircraft engines in a gas stream with a temperature of about 1700 °C and a centrifugal force of the order of tons [1]. They are made of CMSX-4 superalloy characterised by excellent mechanical and strength properties, especially at high temperatures. The alloy contains 10 major alloying elements, and its chemical composition is as follows (in wt %): Ni–9.5 Co–6.4 Cr–6.4 Ta–6.4 W–5.6 Al–2.9 Re–1.0 Ti–0.6 Mo–0.1 Hf [1]. Currently, these alloys are used most often on production lines. During production, at the stage of directional dendritic crystallisation using the Bridgman technique, obtained blade casts contain a set of parallel dendrites with the same crystal orientation of the [001] type. This ensures that the blades have high creep resistance at high temperatures [1]. The orientation is given to the cast by a selector with a narrow diameter, which is cut off at the next stage of production. The selector is connected to a wide root. During crystallisation, change in the cross-sectional area where crystallisation occurs from the narrow selector to the wide root causes the formation of various defects such as low-angle boundaries (LABs) [2,3], vacancies [4], and casting stress [5,6]. These root defects are inherited by airfoil during crystallisation of the blade and often do not disappear even after heat treatment is applied on production lines [7]. Therefore, studies of LABs in the research of the root are very important.

Low-angle boundaries and casting stresses may cause delamination and crack of the protective coatings of blades during implementation [8], thus reducing their service life. The cross-sectional area of the root is much larger than the airfoil. In the areas of the root with macroscopic low-angle boundaries (LABs), the concentration of vacancies and their clusters in the γ′-phase is increased [6]. Therefore, it can be assumed that near the LABs, changes in the lattice parameter of the γ′-phase (hereinafter referred to simply as *a*_γ′_) may occur.

The X-ray diffraction topography methods used to study single-crystalline blades are very useful for characterising macroscopic defects related to local changes in crystal orientation, such as LABs [3,6,9]. Based on X-ray topograms of single-crystalline blades, crystal misorientation of adjacent dendrites or their groups and low-angle boundaries (LABs) can be visualised. A value of misorientation angle may also be defined by the method described in Ref. [10]. Additionally, the diffraction contrast that creates the topograms also contains information about the change in the lattice parameter. In Ref. [11], it is shown that the lattice parameter of the γ′-phase of the SX CMSX-4 superalloys near the LABs is decreased. However, precise measurements of the lattice parameter near the LABs have not been made. In Ref. [3], X-ray reflection topography of two surfaces of lamellar samples was investigated for the first time. It was found that the image of misorientation defects (i.e., LABs) from both sample surfaces, having the size of the sample thickness, is characterised by the contrast inversion in the topograms. However, a detailed analysis of the angles of misorientation determined for the topograms from both surfaces has not been performed.

It is generally assumed that in the X-ray topograms of SX superalloys, two types of macroscopic LAB contrast can be distinguished. The first is related to the abrupt crystal lattice rotation of local macroscopic regions called the subgrains. This rotation is characterised by the angle of misorientation. The second is related to a local change in the interplanar spacing of crystal lattices near the LAB. These changes may be related to the bending of the dendrites [12,13] or a local change in the chemical composition of the cast. 

The superalloy cast consists mainly of two phases, γ and γ′, at room temperature. The creation of LABs and areas of lattice parameter changes occurs during dendritic crystallisation of the γ-phase in a complex-shaped casting mould. After crystallisation, both in the dendrites and the interdendritic regions, the γ-phase changes according to the transition γ_I_ → γ′ + γ_II_, where γ_I_ has a chemical composition corresponding to the mushy zone of Ni-Al phase equilibrium diagram, and γ_II_ corresponds to the γ and γ′ equilibrium region of this diagram. In the vicinity of the LAB, changes in the lattice parameter *a*_γ′_ may occur due to inheriting the original γ_I_ lattice parameter changes. These changes in lattice parameters are related to the heterogeneity of the chemical composition resulting from the segregation of numerous alloying elements both on the dendrite scale and on the γ/γ′ structure scale. The LABs in CMSX-4 are located in the interdendritic regions, where the chemical composition of the superalloy is different from the nominal composition of CMSX-4 [14,15]. It is related to the so-called dendritic segregation of alloying elements during the directional crystallisation process [16,17]. In the airfoil of the blades, dendritic segregation has a very complex nature, as the set of parallel dendrites crystallises in a reasonably narrow area between the suction and pressure surfaces of the airfoil. The changes in the chemical composition and *a*_γ′_ heterogeneity around the LABs, in this case, can have complex mechanisms related to the crystallisation of the dendrites near the surface of the casting mould [18]. However, in the bulk root, the side surfaces are mostly parallel to the direction of crystallisation. Therefore, to examine the relationship between LAB angles of misorientation and lattice parameter *a*_γ′_ changes near the LABs, the blade roots were chosen. The interaction of dendrites with the walls of the mould is much smaller than in the airfoil. Therefore, the relationship between the LAB and the lattice parameter of the γ′-phase is devoid of the influence of these walls. The interpretation of the measurement result should be significantly simplified. Additionally, the measurement of *a*_γ′_ by X-ray diffraction methods is relatively simple as the content of this phase in CMSX-4 superalloy is high and amounts to 70% [1], so its contribution to the X-ray diffraction pattern is large.

The aim of the study is to determine the relationship between the misorientation angles of LABs and changes in the lattice parameter of the γ′-phase near the LABs in the roots of single-crystalline blades made of the CMSX-4 alloy.

## 2. Materials and Methods

The single-crystalline blades were produced at the Research and Development Laboratory for Aerospace Materials at the Rzeszów University of Technology, Rzeszów, Poland. The specimens were prepared from the roots of CMSX-4 turbine blades obtained with a 3 mm/min withdrawal rate by the Bridgman method. 

The sample studies were carried out using X-ray diffraction topography and the measurement of the lattice parameter *a*_γ′_ by the EFG Ω-scan X-ray diffraction method using the EFG Freiberg Instruments X-ray diffractometer (Freiberg Instruments, Freiberg, Germany) [19]. The dendritic structure was visualised by scanning electron microscopy (SEM) with backscattered electron imaging (BSE). The JSM-6480 JEOL electron microscope (JEOL Ltd., Tokyo, Japan) was used to visualise the dendrite array. The crystal orientation of the samples was determined in back-reflection geometry by the Laue diffraction using the X-ray diffractometer of an XRT-100 system provided by EFG Freiberg Instruments. 

**Figure 1:**
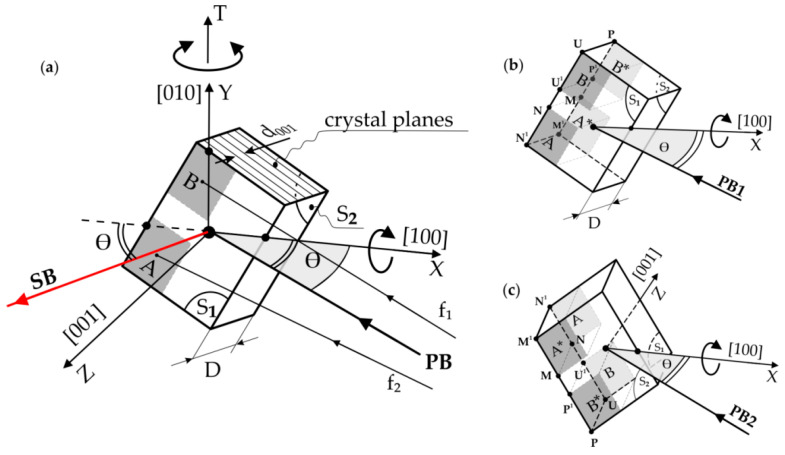
Scheme of the X-ray diffraction topography method with oscillation about the T-axis of the diffractometer, which is consistent with the Y-axis of a plate-shaped sample (**a**) and sample settings during X-ray topogram recording from surfaces S_1_ (**b**) and S_2_ (**c**). The X and Y axes lie in the sample surface S_1_, and the Z-axis is perpendicular to it. d_001_—an interplanar spacing. PB and SB—the fragments of the primary and secondary (diffracted) beam. *f*_1_ and *f*_2_—the divergent fragments of PB.

The studies used X-ray reflective diffraction topography with oscillation of the samples and the X-ray film around the Bragg angle. The divergent X-ray beam of characteristic Cu_Kα_ radiation was applied using the PANalytical Microfocus DY0601 diffractometer (Malvern PANalytical, Almelo, The Netherlands). During topogram recording, the divergent beam coming from a quasi-point X-ray source illuminates the whole surface of the sample. The topograms were obtained using 002 type of reflexes coupled with an X-ray film sample oscillating about the vertical axis T by an angle of ±4° about the Bragg angle θ (Figure 1). Topograms were recorded on AGFA Structurix D7 films. 

The plate-shaped specimens, with a small thickness of *D* = 0.5 mm and surfaces S_1_ and S_2_, perpendicular to the direction [001]_γ/γ′_, were prepared from the blade root (Figure 1). The tested surfaces were prepared according to the procedures for preparing the specimens necessary for X-ray measurements [20]. X-ray topograms were recorded from both surfaces S_1_ and S_2_.

In the first stage, the sample was mounted on the goniometer head of the diffractometer in such a way that the crystal direction [010], parallel to the Y-axis of the sample (Figure 1), was arranged parallel to the vertical axis T of the diffractometer. The direction [100] of the sample was parallel to the X-axis and horizontal. The divergent primary beam (PB) covered the entire surface S_1_ of the sample. In Figure 1, only the central fragments and two divergent fragments *f*_1_ and *f*_2_ of the primary and central fragments of the secondary beam (SB) are shown for clarity. The distance of the radiation source from the sample surface (about 500 mm) was much greater than the sample size (20 mm × 15 mm). During the oscillation of the sample, various fragments of the surface S_1_ successively fulfil the Bragg condition, and a topogram of the entire surface S_1_ is gradually recorded on the film. After recording the topogram from the S_1_ surface, the sample was rotated about the X-axis by 180°, and the X-ray topogram from the S_2_ surface was then recorded. 

**Figure 2:**
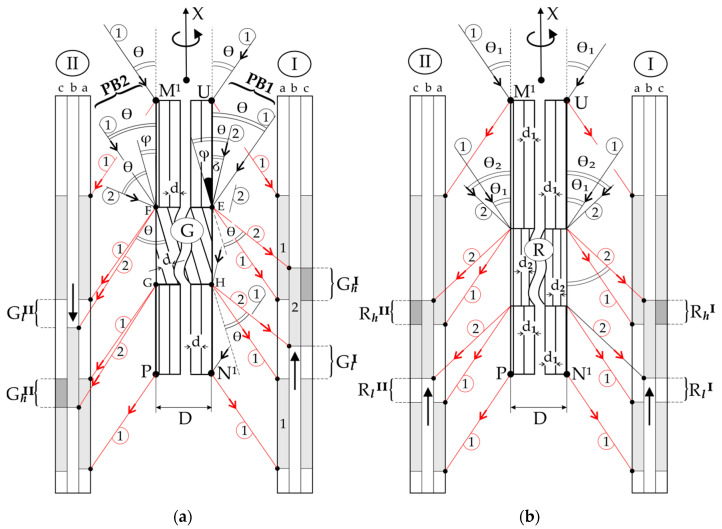
Schemes of contrast inversion of the G area with a lattice rotation by the angle φ (**a**) and the lack of contrast inversion in the case of the R area with a changed interplanar spacing *d* (**b**). Black arrows correspond to the fragments of the primary beam, and red arrows correspond to the secondary (diffracted) beam fragments. The φ is enlarged to figure clarity. I and II—marked the two sets of X-ray film and primary beam placement.

Let us consider the hypothetical case of X-ray diffraction from two surfaces of the thin sample, where, in the area G, there is the rotation of the crystal lattice by the angle φ relative to the rest of the sample (Figure 2). Additionally, the interplanar spacing *d* of diffraction planes of the G area is the same as outside of this area. The diffraction planes of the (001) type are parallel to the S_1_ and S_2_ surfaces of the sample, and the size of G is of the order of the thickness *D* of the sample (Figure 1). In order to reduce the number of drawings, Figure 2a shows two diffraction geometries as if there were two primary beams PB1 and PB2 and two X-ray film placements, I and II. 

These two diffraction geometries may be named as two sets of PB and film placement. Set I with PB1, which corresponds to the diffraction scheme from the surface S_1_ (Figure 1), is shown in Figure 2 in the top view. In contrast, set II with PB2 corresponds to the diffraction scheme from the surface S_2_ (Figure 2) in the bottom view. The axis T (Figure 1) of coupled film and sample oscillation is perpendicular to the surface of Figure 2.

These two sets can be realised when we rotate the sample about the X-axis by 180°, with a constant position of the primary beam, marked as PB1 or PB2 in Figure 1. 

It was assumed that the size of area G is so large that it is “visible” on both surfaces S_1_ and S_2_ (Figure 1), marked in Figure 2 as UN^1^ and PM^1^. In both cases, the divergent primary beam covers the surface completely. At a certain arrangement of coupled sample and X-ray film oscillation, some fragments of the primary beam satisfy the Bragg condition for the E point of the upper sample surface part UE. This fragment is deflected in the direction of the secondary beam 1 (red arrow 1). The points of the UE part satisfy the Bragg condition at different times when the various parts of the primary beam fall on it at the Bragg angle. In this way, the image of part UE is gradually recorded on the hypothetical layer *a* of the X-ray film 1 (Figure 2a). The recording of the image topogram of part HN^1^ on the hypothetical layer “*a*” is similar. This is performed by reflecting various fragments of the primary beam at the same angle (Bragg) taking place at different moments in the sample and X-ray film oscillation.

During the oscillation of the sample around the T (Y) axis (Figure 1), for a certain range of sample oscillation angles, some fragments of the PB1 beam, marked as black arrows 1 in Figure 2a, met the Bragg condition for various parts of the UN^1^ surface, except for the part EH (Figure 2a) corresponding to the G area of the sample. Different fragments of the primary beam (black arrows 1) deflect on various points of the upper and lower parts of the UN^1^ surface, creating diffracted beam fragments that are marked with red arrows 1. At this range of oscillation angles of the sample on the X-ray film, the exposition of the 1st part of hypothetical layer “*a*” of the film is created. As a result of exposition, the contrast of these fragments increases, i.e., its blackening is gradually increased. 

Where PB1 fragments are reflected from the region G, the angle of its incidence must be equal to the angle δ = θ − φ (Figure 2a). The angle δ is shown in Figure 2a for point E, common to area G and upper region UE of the sample. The angle δ is constant for the entire surface EH of area G. The fragments of the diffracted beam are shown in Figure 2a for the surface of the G area in the form of red arrows 2. The blackening of the 2nd part of hypothetical layer *b* of the X-ray film is created at different sample oscillation angles. Based on Figure 2a, it can be seen that the image of area G on the hypothetical layer *b* of the X-ray film will be shifted upwards. On the real X-ray film, the blackening of both hypothetical layers *a* and *b* overlap, creating a topogram, the contrast of which is shown on the *c* layer. As a result of the above-described overlap, an area of increased contrast G_h_^I^ causes increased contrast on the X-ray film, and an area of lowered G_l_^I^ contrast, which causes decreased contrast on the X-ray film, will be formed on the real topogram. Similar reasoning can be made for the situation in which the primary beam PB2, which some fragment marked as 1 in Figure 2a, covers the surface of PM^1^ corresponding to the S_2_ surface in Figure 1. For set II in Figure 2a, not all fragments of the primary beam are shown. In this case, on the hypothetical layer b of the film, the image of the area G will be shifted downwards, resulting from which areas of increased G_h_^II^ and lowered G_l_^II^ contrast will be created on layer *c*, i.e., on the topogram. When we compare the topograms obtained from the two surfaces of the sample, instead of the high-contrast area G_h_^I^ obtained with set I on the topogram, there will be a low-contrast area G_l_^II^ obtained with set II. Similar relationships exist for the areas G_l_^I^ and G_h_^II^. Comparing the topograms (layers *c*) obtained with sets I and II, the inversion of the contrast originating from the same defect of misorienting character can be observed. This type of contrast inversion was first presented in Ref [7].

Figure 2b shows a scheme of diffraction, wherein in a certain area R of the sample, there is a reduction in the interplanar spacing (*d*_2_ < *d*_1_), without the crystal lattice rotation. For the upper and lower sample fragments with interplanar spacing *d*_1_, the Bragg angle is θ_1_. However, in this case, the deflection of X-ray according to Bragg conditions will take place at a greater θ_2_ angle for the R area. The scheme shows that the image shift of this area on the hypothetical layer *b* of the X-ray film will be for both surfaces UN^1^ and PM^1^ in the same direction—upward. Therefore, for sets I and II for the area R, an increased contrast of R_h_^I^ and R_h_^II^ will be created in the same place of both topograms, i.e., on layers *c*. The low-contrast areas R_l_^I^ and R_l_^II^ will also be in the same place, so there will be no contrast inversion.

## 3. Results and Discussion 

**Figure 3:**
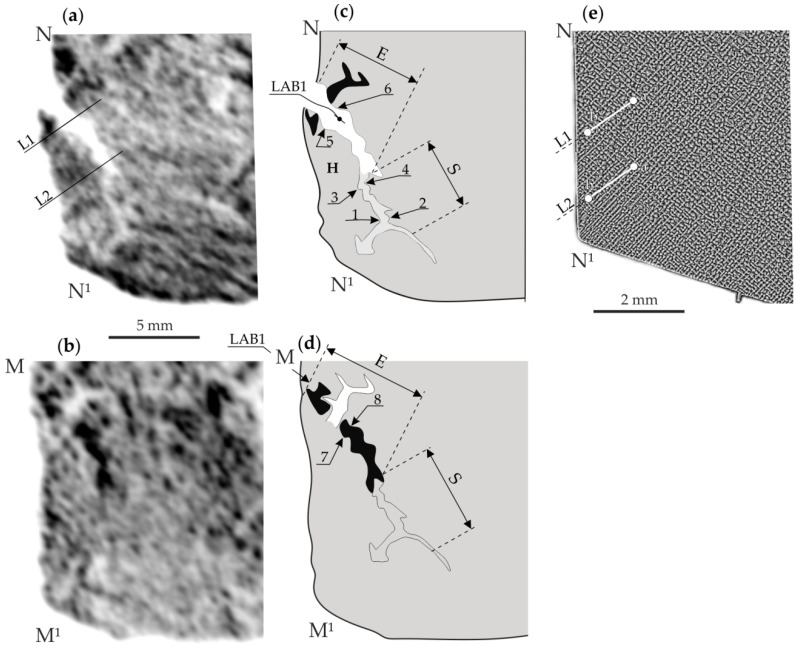
Examples of X-ray topograms obtained from fragment A of surface S_1_ and fragment A* of the S_2_ surface (**a**,**b**) with their schemes (**c**,**d**) and the dendritic structure visualised on surface S_1_ (**e**). Since the topogram from fragment A* was obtained from the mirror surface of fragment A, it was transformed by mirroring for easier comparison of areas of the same LAB visualised on both surfaces S_1_ and S_2_.

**Figure 4:**
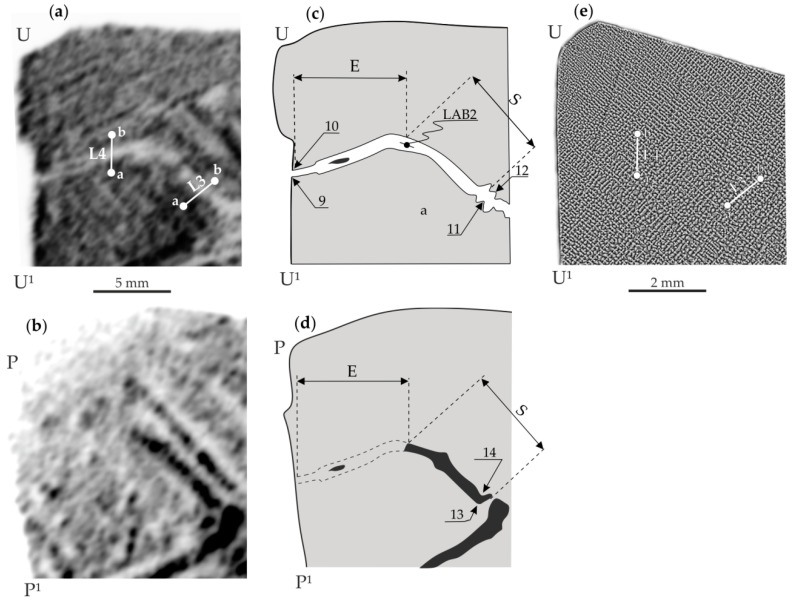
Examples of X-ray topograms obtained from fragment B of surface S_1_ and fragment B* of surface S_2_ (**a**,**b**) with their schemes (**c**,**d**) and the dendritic structure visualised on the surface S_1_ (**e**). Since the topogram from fragment B* was obtained from the mirror surface of fragment B, it was transformed by mirroring for easier comparison of areas of the same LAB visualised on both surfaces S_1_ and S_2_.

Figure 3 and Figure 4 show representative examples of X-ray topograms of the blade root fragments with the schemes of the topograms and the dendritic structure visualised on the S_1_ surface. 

Figure 3a,b shows the topograms obtained from the fragments NN^1^ and MM^1^ obtained from fragment A of surface S_1_ and fragment A* of the S_2_ surface. Low-angle boundary 1 (LAB1) is clearly visible in Figure 3a as a serpentine strip of decreased contrast. Additionally, part of LAB1 in the form of inverted increased contrast is shown in Figure 3b. LAB1 schemes are shown in Figure 3c,d. Figure 3e shows the image of the dendritic structure of the area from which the topogram presented in Figure 3a was obtained. The analysis of the dendritic structure does not allow perceiving the shape of LAB1. The image LAB1 in the topogram in Figure 3a was created by shifting fragment H (Figure 3c) about the rest of the topogram. Based on the analysis of Figure 3a, it can be concluded that the initial parts of the LAB1 distance, determined from the edge of the sample near the point N of the local shift to the width of the bright serpentine band in the topogram, is small. The final part E (Figure 3c), near the edge of the sample of fragment A, is greater. This means that the initial part’s misorientation angle is small, and the ending part E is greater. Comparing the images of LAB1 in Figure 3a,b, it can be concluded that the initial part S visible in the topogram in Figure 3a (scheme in Figure 3c) is not visible on the topogram in Figure 3b. 

Additionally, in the topogram obtained from fragment A* (Figure 3b), the width of the dark strip of part E compared to the width of the bright strip of part E visible in Figure 3a (obtained from fragment A) is generally smaller. Using the method described in Refs [3,4,21], Appendix A and based on shifts of characteristic points 1 and 2, 3 and 4, and 5 and 6 of the topogram in Figure 3a, obtained from fragment A of the surface S_1_, the misorientation angles corresponding to these points were determined: α_12_ = 0.07°, α_34_ = 0.18°, and α_56_ = 0.43°, respectively. However, for the topogram in Figure 3b obtained from fragment A* of the surface S_2_, only the LAB1 local misorientation angle for points 7 and 8 was calculated: α_78_* = 0.11°. 

Figure 4a,b shows the topograms obtained from fragment B of surface S_1_ and fragment B* of surface S_2_ of another sample cut from the blade root. In Figure 4a (obtained from fragment B), the low-angle boundary 2 (LAB2, scheme in Figure 4c) is visible in the form of a bright band that changes the orientation roughly in the point of E and S parts’ connection (Figure 4c). However, in Figure 4b (obtained from fragment B*), the beginning of part S of LAB2 is visualised as a dark band, and the image of its end (part E) is not visible. Using the topograms in Figure 4a,c, the local misorientation angles for LAB2 were calculated for characteristic points 11 and 12, as well as for 9 and 10: α_11,12_ = 0.39°, α_9,10_ = 0.08°, respectively. Additionally, for the topogram in Figure 4b recorded from the surface S_2_, the α*_13,14_ value was calculated, which was 0.32°. Comparing the topograms in Figure 3 and Figure 4 shows that images of the S part of LAB1 and the E part of LAB2 are not visible on the topogram obtained from the surface S_2_ of the samples. 

**Figure 5:**
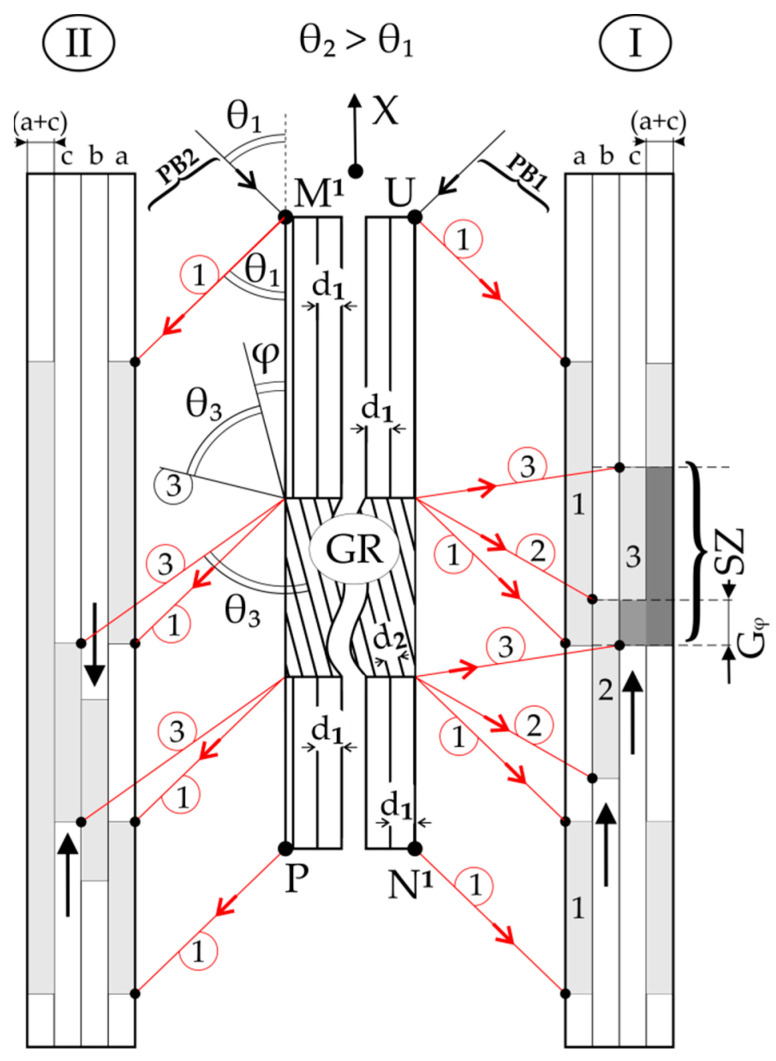
Scheme of contrast creation on two surfaces of a lamellar sample, where the crystal planes of the GR area are rotated by the angle φ and the spacing of their diffraction planes *d*_2_ is smaller than in the rest of the sample. For clarity of the figure, only some primary beams are marked. φ angle is also enlarged to figure clarity.

On the other hand, the images of part E of LAB1 (Figure 3a) and part S (Figure 4a) of LAB2 obtained from the surfaces S_1_ and S_2_ are related by contrast inversion. In other words, low-contrast areas have been converted to high-contrast areas. This effect corresponds to the scheme shown in Figure 2a. However, the disappearance of a part of the LABs image may be related to the interplanar spacing of crystal lattices. The mechanism of this phenomenon can be explained based on the topogram contrast creation scheme shown in Figure 5. In this figure, the diffraction planes of the GR area with a spacing *d*_2_ smaller than *d*_1_ are rotated according to the rest of the sample by a misorientation angle φ. 

Similar to the case described above in the Materials and Methods section (Figure 2 and its description), the deflection of fragment 1 of the primary beam PB1 from the surface of the GR area occurs at a different angle than the Bragg angle of the rest of the surface UN^1^. During the oscillation of the sample, for a certain range of oscillation angles, the Bragg condition is satisfied for the entire surface UN^1^, except for the fragment corresponding to the GR area of the sample. For this range of sample oscillation angles, on the hypothetical layer “*a*” of X-ray film, the increased contrast of the first parts is recorded. At a different range of the sample oscillation angles, a shift in the second part of hypothetical layer “*b*” is created. This part is related to the rotation of the diffraction plane by the angle φ. If *d*_2_ is equal to *d*_1_, then as a result of the overlap of layers 1 and 2, the area G_φ_ of increased contrast is created in the topogram (layer “*c*”). Additionally, considering condition *d*_2_ < *d*_1_, the Bragg angle θ_3_ of the GR area will be greater than θ_1_ of the remaining sample areas. This condition causes the additional upward shift of the third contrast part of the hypothetical layer “*c*”. The third contrast part of the hypothetical layer “*c*” will take the position determined by the shift due to the rotation of the crystal planes of the GR area by the angle φ and additionally by the shift related to the increase in the Bragg angle of the GR area. In this case, area SZ of increased contrast will appear in the topogram (Figure 5).

On the topogram obtained from the surface UN^1^ (S_1_), the contrast of the SZ area resulting from the overlapping the effect of crystal misorientation and the effect of lowering the value of *d* (*d*_2_ < *d*_1_) will be expanded and compared to the contrast G_φ_ as a result of crystal misorientation only. However, in the topogram from the S_2_ surface, the contrast may not occur at all due to shifts in contrast of the GR area in opposite directions. This case is illustrated in Figure 5 for set II and primary beam PB2. The downward shift of the contrast from GR on the hypothetical layer *b* is related to the rotation of the GR lattice by the angle φ. This shift can be completely compensated by an upward shift related to decreasing *d* and consequently increasing the Bragg angle to the value θ_3_. Then, on the real topogram, which is the sum of the images of the hypothetical layers “*a*” and “*c*”, there will be no GR contrast shifts at all. In this case, in the topogram from the surface S_2_ of the sample, the contrast from the LAB between GR and the rest of the sample is not created.

To verify that the disappearance of a part of the LAB image on the topograms from the S_2_ surface is related to the change in *d*, the lattice parameter *a*_γ′_ of the γ′-phase was measured for the sections a-b of two lines L1 and L2 passing through LAB1 (Figure 3a,e) and for two sections of the lines L3 and L4 passing through LAB2 (Figure 4a,e). It was assumed that the diffraction contrast on the topograms of the CMSX-4 superalloy blade roots was created by the γ′-phase because the share of this phase compared to the γ-phase is significantly higher (about 70% by volume). 

**Figure 6:**
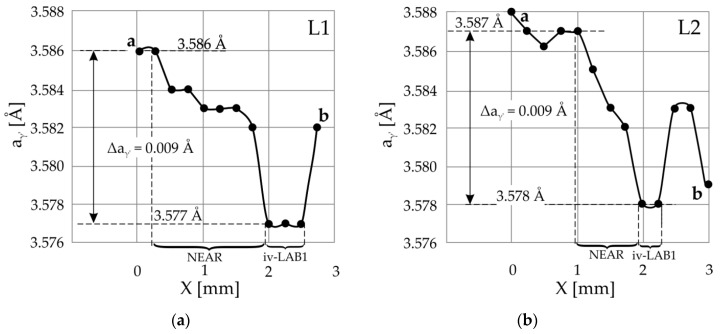
Distribution of the lattice parameter *a*_γ′_ of the γ′-phase for Sections a-b along with Line L1 (**a**) and the line L2 (**b**) passing in the immediate vicinity of LAB1 (iv-LAB1). The arrangement of lines L1 and L2 is shown in Figure 3a,e.

**Figure 7:**
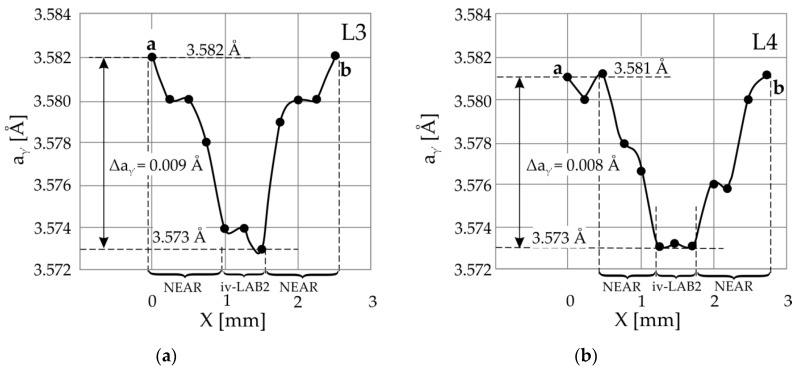
Distribution of the lattice parameter *a*_γ′_ of the γ′-phase for the sections a-b along with the line L3 (**a**) and the line L4 (**b**) passing in the immediate vicinity of LAB2 (iv-LAB2). The arrangement of lines L3 and L4 is shown in Figure 4a,e.

Figure 6a,b and Figure 7a,b shows the distribution of lattice parameter *a*_γ′_ along two lines L1 and L2, passing in the immediate vicinity of LAB1, and along two lines L3 and L4, passing in the immediate vicinity of LAB2. The graphs show that near LAB1, there is a significant decrease in *a*_γ′_ and hence a decrease in the interplanar spacing *d*.

For the L1 line in the immediate vicinity of the LAB1 boundary, the lattice parameter value is in the order of 3.577 Å and far from it, where the low-angle boundary is not clearly visible in the topogram is in the order of 3.586 Å. Near point *a* (Figure 6), the lattice parameter *a*_γ′_ changes stochastically around 3.586 Å. Therefore, the change in the lattice parameter *a*_γ′_ near the area iv-LAB1 region is 0.009 Å for all their characteristic points 1–6. The *a*_γ′_ values presented in Figure 6a,b on the right side of area iv-LAB1 correspond to the points located near the edge of the sample corresponding to the root’s surface. They can be disturbed by a change in the crystallisation conditions near the wall of the casting mould in contact with the areas located far from the root surfaces. Using the Bragg equation, it can be calculated that the change in the Bragg angle ∆θ for the 002 Cu_Kα_ reflex corresponds to the change in the ∆*d* = 0.009 Å is 0.06°. Based on significant changes in the lattice parameter *a*_γ′_ in the immediate vicinity of LAB1, it can be concluded that the lattice of the γ′-phase is stressed. The disappearance of a part of the image of LAB1 on the topograms obtained from the surface S_2_ according to the scheme presented in Figure 5 can be explained by decreasing the shifts of contrast in topogram related to the reduction of the interplanar spacing *d*. The following calculations confirm this.

Calculated from the topogram on the surface S_1_, the misorientation angle α_34_ of the LAB1 area located near points 3 and 4 is 0.18°, whereas the α*_78_ corresponding to the same LAB1 area, calculated from the topogram of the S_2_ surface, is α*_78_ = 0.11°. The difference α_34_ − α*_78_ = 0.07° is very close to the value Δθ = 0.06° related to the change in the lattice parameter *a*_γ′_. It should be assumed that between the misorientation angles specified for the surfaces S_1_ and S_2_, there will be a relationship:

α* = α − Δθ, (1)

where α* is the misorientation angle defined from the topogram obtained from the surface S_2_, and α is the misorientation angle defined from the topogram obtained from the surface S_1_. Δθ is a change in the lattice parameter *a*_γ′_.

For the value of α_12_ = 0.07° using Equation (1), it was found that the angle of misorientation calculated for the same LAB1 area using the topogram obtained from the S_2_ surface will be almost equal to “0” (0.07° − 0.06° = 0.01°). Therefore, the contrast of the LAB1 area near points 1 and 2 will not be visible on the topogram from the S_2_ surface.

Based on the graphs presented in Figure 7, the change in the Bragg angle related to the lattice parameter changes in the immediate vicinity of the LAB2 was also determined. The mean value of Δθ, in this case, is 0.06°. For the LAB2 area near points 9 and 10, the value of α_9,10_ is 0.08°. Therefore, the angle α*_9,10_, which would correspond to the topogram on the surface S_2_ according to Formula (1) α* = α − 0.06°, should be equal to: 0.08° − 0.06° = −0.02°. This value differs from zero, although fragment E of LAB2 is not visible on the topogram obtained from the S_2_ surface. This may be related to the location of points 9 and 10 close to the root’s surface, where the displacement of points 9 and 10 was influenced by the interaction of the root with the walls of the casting mould. Unfortunately, in the image of fragment E of LAB2, there are no other characteristic points apart from points 9 and 10. A comparison of the values of α_11,12_ (0.39°) with α*_13,14_ (0.32°) obtained for the same area of LAB2 shows that the difference (α_11,12_ − α_13,14_ = 0.07°) is very close to the value of the changing Bragg angle Δθ (equal 0.06°) related to the change in the interplanar spacing *d*. 

The lattice parameter *a*_γ′_ changes in the immediate vicinity of the LABs shown in Figure 6 and Figure 7 have very distinctive features. In areas with a width of 0.5–1 mm, designated as iv-LAB1 and iv-LAB2, value *a*_γ′_ is almost constant and the smallest. However, near these areas, there are 1–1.5 mm wide “NEAR” areas. In these areas, the lattice parameter *a*_γ′_ decreases when approaching the LAB, which may be related to the casting stresses probably located in those. Additionally, they may be probably related to the change in the concentration of alloying elements or to dendrite bending near the LAB. According to Ref. [6], it may also be related to the formation of vacancy clusters in areas containing low-angle boundaries, which may be based on diffusion of vacancies to dislocations and the Kirkendall effect of alloying elements in γ-phase, which was described for nickel-based superalloys in Ref. [22]. In Ref. [23], it was additionally found that the width of the γ-phase channels varies in the range of 40–100 nm. In our paper [6], it was found that the LAB passes through the γ-phase and “writhes” between the cubes of the γ′-phase with a size of about 1 µm. However, in our present study, the width of the LAB is in the order of 1 mm, and the width of the “NEAR” regions is approximately 1–1.5 mm in total. This is probably related to the fact that the LAB “writhes” in the area between the arms of the dendrites at the primary arm spacing of the order of hundreds of µm and the adjacent dendrites “writhes” by deviating from each other by millimetres. This morphology of LABs confirms the serpentine strip of LAB1 in the topogram shown in Figure 3a.

## 4. Conclusions

(1) The X-ray diffraction topography method used for the first time on two surfaces of thin lamellar samples coupled with the lattice parameter measurements near the LAB may allow separating the misorientation component of diffraction contrast from the component that arises as a result of lattice parameter changes. The last component of topography contrast allows the creation of a stress distribution map of single-crystalline blades made of superalloys in the aerospace industry. This, in turn, will allow identifying the areas of possible delamination of the protective layers applied to the blades.

(2) For the first time, it was found that there are two areas of the decreased lattice parameter of the γ′-phase, as follows:

- In the immediate vicinity of the low-angle boundaries, the lattice parameter *a*_γ′_ in the area about 0.5–1.0 mm wide is lowered by 0.008–0.009 Å in relation to areas distant from the LAB.
- Additionally, near the low-angle boundaries, there are 1–1.5 mm wide areas (“NEAR” areas) in which lattice parameter *a*_γ′_ decreases when approaching the LAB. Based on local changes of the lattice parameter, it can be concluded that the internal stress might be located in these areas. They may be related to the change in the concentration of alloying elements or/and dendrite bending.

## Figures and Tables

**Figure A1:**
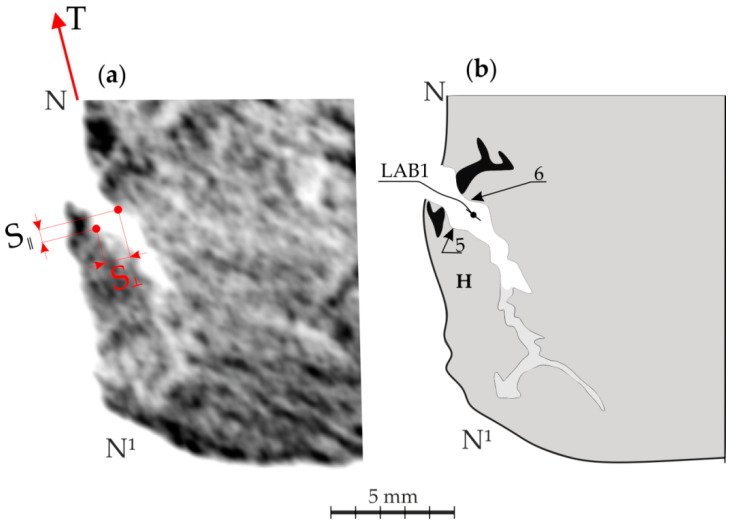
Exemplary X-ray topogram with marked for calculations components S_┴_ and S_║_ (**a**) and its scheme with marked points 5 and 6 (**b**). S_║_—displacement in the direction of oscillation axis T, and S_┴_—displacement along axis X perpendicular to T.

## Data Availability

Data sharing is not applicable to this article.

## Appendix A

An example of calculating misorientation angle for points 5 and 6.

Based on the topogram presented in Figure A1 (simplified topogram of Figure 3a), the components α_x_ and α_z_ of misorientation angle for LAB1 were calculated using Formulas (A1) and (A2) [10]:

(A1) $$\sin\left(\alpha_{x}\right)=\frac{1}{2\sin\theta_{002}}\cdot \frac{S_{∥}}{\sqrt{S_{∥}^{2}+R^{2}}}$$

(A2) $$\sin\frac{\alpha_{z}}{2}=\frac{S_{┴}}{2R}\cdot \sin\left(\theta_{002}\right)$$

where *S_║_* equal to 0.51 mm and *S_┴_* equal to 1.04 mm are the shifts along the X and Z axes measured in the topogram, *θ*_002_ is the Bragg angle equal to 25°, and R is the sample film distance equal to 100 mm. The total misorientation angle for LAB1 was calculated based on Formula (A3):

(A3) $$\alpha =\sqrt{\alpha_{x}^{2}+\alpha_{z}^{2}}$$

where *α_x_* and *α_z_* are equal to 0.35° and 0.25°, respectively. The calculated value of α for LAB1 was 0.43°. The misorientation angles for the remaining points visible in Figure 3 and Figure 4 were calculated similarly.
